# Musculoskeletal complaints, working conditions, and job satisfaction among Dutch orthodontists

**DOI:** 10.1093/ejo/cjag046

**Published:** 2026-06-10

**Authors:** Annemieke Bos, Ronald M van der Bie, David B Hardjosantoso, Ronald E G Jonkman, Jan J M Bruers

**Affiliations:** Academic Centre for Dentistry Amsterdam, Department of Orthodontics, University of Amsterdam and Vrije Universiteit Amsterdam, Gustav Mahlerlaan 3004, Amsterdam 1081 LA, The Netherlands; Academic Centre for Dentistry Amsterdam, Department of Orthodontics, University of Amsterdam and Vrije Universiteit Amsterdam, Gustav Mahlerlaan 3004, Amsterdam 1081 LA, The Netherlands; Academic Centre for Dentistry Amsterdam, Department of Orthodontics, University of Amsterdam and Vrije Universiteit Amsterdam, Gustav Mahlerlaan 3004, Amsterdam 1081 LA, The Netherlands; Academic Centre for Dentistry Amsterdam, Department of Orthodontics, University of Amsterdam and Vrije Universiteit Amsterdam, Gustav Mahlerlaan 3004, Amsterdam 1081 LA, The Netherlands; Academic Centre for Dentistry Amsterdam, Department of Oral Public Health, University of Amsterdam and Vrije Universiteit Amsterdam, Gustav Mahlerlaan 3004, Amsterdam 1081 LA, The Netherlands

**Keywords:** orthodontics, musculoskeletal complaints, working conditions, job satisfaction

## Abstract

**Background:**

This study explored the extent to which orthodontists experience work-related musculoskeletal disorders (WMSDs) and burdensome working conditions, assessed job satisfaction, and identified factors associated with job satisfaction.

**Methods:**

Data were collected by means of a web-based survey comprising 64 items addressing general and professional characteristics, work-related musculoskeletal complaints, and five subscales addressing working conditions, stress, private circumstances, coping ability, and job satisfaction. Descriptive statistics, reliability analyses, and bivariate and multivariable linear regression analyses were performed.

**Results:**

In total, 115 orthodontists participated. Almost three-quarters (73.0%) reported at least one WMSD, with the shoulders, neck, and lower back being the most frequently affected anatomical regions. While orthodontists reported a moderate burden from working conditions and private circumstances, most experienced low levels of stress, demonstrated high coping ability, and reported high job satisfaction. In bivariate analyses, fewer WMSDs, lower perceived burden from working conditions and stress, and higher coping ability were associated with greater job satisfaction. In the multivariable model, working conditions, practice ownership, and coping ability explained 25.2% of the variance in job satisfaction.

**Conclusion:**

Greater job satisfaction was most strongly associated with more favourable working conditions, owning a practice, and higher coping ability. Although lower perceived stress and fewer work-related musculoskeletal disorders were associated with job satisfaction in unadjusted analyses, they did not remain independent predictors in the multivariable model. These findings suggest that both organizational and individual factors contribute to professional satisfaction among orthodontists. However, additional determinants not examined in this study are likely to play a role.

## Introduction

A substantial proportion of orthodontists in the Netherlands will retire in the near future [1]. This outflow is expected to exceed the inflow of newly qualified orthodontists from the university training programmes. Whether this imbalance will result in a shortage of orthodontic capacity depends on developments within the profession, changes in the field of practice, and the demand for orthodontic care. A shortage of capacity may compromise the quality of treatment results, either due to insufficient time per patient or as a consequence of elevated work-related stress among orthodontists [2]. Among dentists, stress and burnout have been reported as the principal reasons for leaving the profession [3]. For the recruitment and retention of orthodontists, job satisfaction appears to be of great importance [4].

These findings raise the question of how Dutch orthodontists experience their professional practice. General dentistry, a closely related profession, is widely regarded as a stressful occupation [5]. The primary sources of occupational stress include time and scheduling pressures, professional concerns, patient and public perceptions of dentists, staffing difficulties, pressures associated with patient treatment, and administrative or business-related demands. Prolonged exposure to stress may have adverse physical, emotional, and social consequences. Numerous studies examining the stressful aspects of dental practice have concluded that work-related stress is negatively associated with job satisfaction, physical health, and mental health [6, 7]. Nevertheless, general dental practitioners are generally reported to be satisfied with their work [4, 8].

In addition to its negative association with job satisfaction, stress has been linked to physical conditions, such as work-related musculoskeletal disorders (WMSDs) and coronary heart disease, as well as psychosocial conditions, including burnout [9–12]. Dentists are at increased risk of developing WMSDs owing to prolonged work in unnatural or constrained postures, combined with the need for sustained precision and concentration [13]. Prolonged static postures, resulting in sustained muscle loading of the neck and shoulders, are recognized risk factors for WMSDs [13, 14]. Orthodontists and dentists show comparable prevalence rates of WMSDs [14, 15]. However, the pattern of complaints differs: dentists are more likely to report shoulder complaints, whereas orthodontists more frequently report hand and wrist complaints [16].

Work-related stress may also lead to burnout. Among working dentists, 21% were found to have an elevated risk of burnout, 13% reported high overall levels of burnout, and 2.5% were classified as severely burned out [17]. These findings underline the importance of coping with occupational stress. Participation in stress management courses may substantially improve dentists’ working conditions [18]. Important factors in alleviating stress include remaining within one’s professional comfort zone and expanding this zone by enhancing psychological resilience [19].

Studies conducted among orthodontists have yielded findings comparable to those reported for dentists. Although orthodontists generally report being satisfied with their profession, a negative correlation between occupational stress and job satisfaction has been observed [6, 20]. In orthodontic practice, the most prominent stressors relate to time management and patient cooperation [21]. However, other factors may exert a greater influence on perceived occupational stress than those evaluated in previous research [5]. While stress and job satisfaction have been extensively studied among dentists, research focusing specifically on orthodontists remains limited. Consequently, less is known about the factors associated with occupational stress and job satisfaction within the orthodontic community. In light of an increasing demand for orthodontic care and a declining capacity of orthodontists, insight into current working conditions, stress levels, and job satisfaction among Dutch orthodontists may contribute to maintaining a sustainable profession and to improving patient care and safety in the years ahead [19].

Therefore, the aim of this study was to investigate the extent to which orthodontists experience WMSDs and burdensome working conditions, to assess their level of job satisfaction, and to identify factors associated with job satisfaction among orthodontists.

## Materials and methods

For the reporting of this cross-sectional study, the relevant items of the Strengthening the Reporting of Observational Studies in Epidemiology (STROBE) statement [22] were applied.

### Study design

This study was conducted as a web-based survey and was distributed to all licensed orthodontists in the Netherlands registered with the Royal Dutch Dental Association (Koninklijke Nederlandse Maatschappij tot bevordering der Tandheelkunde, KNMT). In addition to contact details, information on selected general background characteristics (sex, age, and region of residence) was available.

### Research instrument

The questionnaire developed for this study was based on the existing literature and consisted primarily of validated and internationally widely used instruments. It comprised the following components: general and professional characteristics (7 items), work-related musculoskeletal complaints (11 items), psychological complaints (2 items), working conditions (11 items), stress (7 items), private circumstances (5 items), coping ability (11 items), and job satisfaction (10 items).

Work-related musculoskeletal complaints were assessed using the Nordic Musculoskeletal Questionnaire [23]. This instrument includes dichotomous (yes/no) items concerning trouble (ache, pain, or discomfort) in nine anatomical regions: neck, shoulders, elbows, wrists/hands, upper back, lower back, hips/thighs, knees, and ankles/feet. Additional questions addressed whether, during the preceding 12 months, the respondent had been prevented from carrying out normal activities (at home or at work) due to these complaints, and whether such complaints had occurred during the preceding 7 days.

Items relating to working conditions, stress, private circumstances, and dealing with problems, events, or incidents were derived from a study among dentists [7] and from the Utrecht Coping List (UCL) [24]. The UCL is the most frequently used instrument for assessing coping ability in the Netherlands and has demonstrated satisfactory psychometric properties in several validation studies [24–26]. In the present study, a selection of UCL subscales was used, based on their relevance to the physical and psychological workload of orthodontists.

Job satisfaction was measured using the ‘overall job satisfaction’ subscale from a Canadian survey conducted among orthodontists [20], which was originally derived from an American survey developed in the early 1990s. The full instrument comprises 54 Likert-type items, of which 10 items constituting the ‘overall job satisfaction’ subscale were used in this study [8]. For items relating to the degree of influence of a particular circumstance, response options ranged from ‘not’ (1) to ‘very strong’ (5). For items relating to frequency, response options ranged from ‘never’ (1) to ‘very often’ (5).

To reduce acquiescent response bias (acquiescent response set, ARS), the tendency to agree with items regardless of content, both positively and negatively worded statements were included. Face validity of the questionnaire was assessed by five specialists from the Department of Orthodontics at the Academic Centre for Dentistry Amsterdam (ACTA).

### Data collection

An independent research agency distributed an invitation email to all orthodontists in the Netherlands aged 64 years or younger, had a known home and work address in the Netherlands, possessed a valid email address, and were current or former members of the Royal Dutch Dental Association. The email included a personalized link to the web-based questionnaire. Two reminder emails were sent to nonrespondents at 2-week intervals.

### Data processing

The independent research agency processed the returned questionnaires and the available background characteristics (sex, age, and region of residence) in an encrypted database in which individual orthodontists were not identifiable. The anonymized and coded dataset was subsequently provided to the researchers for analysis.

### Statistical analysis

Data were analysed using the Statistical Package for the Social Sciences (SPSS) version 31.0 (IBM Corp., 2025). Descriptive statistics (frequency distributions, means, and standard deviations), reliability analyses, and bivariate and multivariable linear regression analyses were performed. Statistical significance was set at *P* < 0.05.

Reliability analyses were conducted to assess the internal consistency of the scales. Negatively worded items were reverse coded prior to analysis. Cronbach’s alpha coefficients for all subscales (‘Working conditions’, ‘Stress’, ‘Private circumstances’, ‘Dealing with problems and incidents’, and ‘Job satisfaction’) were ≥0.700 and were therefore considered acceptable. Within the subscale ‘Dealing with problems and incidents’, the item ‘using tranquillisers if you feel tense or nervous’ was excluded due to a negative interitem correlation with the remaining items.

The items of the Nordic Musculoskeletal Questionnaire were not treated as a single scale, as complaints in different anatomical regions are not necessarily interrelated. For example, complaints in one body region do not inherently imply complaints in another. However, it was determined whether respondents reported any musculoskeletal complaints.

### Ethical approval

Ethical approval for this study was obtained from the Institutional Review Board of a university dental school. The medical ethics review committee determined that the study was not subject to the national Medical Research Involving Human Subjects Act. The study was conducted in accordance with applicable regulations regarding informed consent and the protection of personal data.

## Results

### General and professional characteristics

Of the 250 orthodontists who met the selection criteria and were invited to participate, 115 (46.0%) completed the survey. Of these respondents, 60.9% were male and 39.1% female. The mean age was 48.5 ± 9.8 years. The majority lived with a partner (90.4%) and had children (73.9%). A substantial proportion (84.3%) reported engaging in sport on a regular basis. The mean duration of professional experience was 18.4 ± 9.7 years. Nearly three-quarters of respondents (73.9%) were private practice owners. On average, respondents worked in practices with five treatment chairs or more (Table 1).

**Table 1:** General and professional characteristics of surveyed orthodontists in the Netherlands.

	*n* (%)	Mean ± SD
Participants	115 (100%)	
Sex		
Male	70 (60.9%)	
Female	45 (39.1%)	
Age in years		48.5 ± 9.8
39 years or younger	25 (21.7%)	
40–49 years	32 (27.8%)	
50–59 years	41 (35.7%)	
60 years or older	17 (14.8%)	
Family composition		
Single	5 (4.3%)	
Single with children	6 (5.2%)	
Partner without children	25 (21.7%)	
Partner with children	79 (68.7%)	
Regularly participating in sports		
Yes	97 (84.3%)	
No	16 (13.9%)	
Unknown	2 (1.7%)	
Professional orthodontic experience		18.4 ± 9.7
Private practice owner		
Yes	85 (73.9%)	
No	30 (26.1%)	
Number of treatment chairs		5.5 ± 1.6
1–2	1 (1.2%)	
3–4	20 (23.5%)	
5–6	43 (50.6%)	
Over 7	19 (22.4%)	
Unknown	2 (2.4%)	

### Work-related musculoskeletal disorders

The majority of orthodontists, 84 of 115 respondents (73.0%), reported experiencing physical complaints in one or more anatomical regions during the preceding 12 months. More specifically, 28.7% reported complaints in a single body region, 28.7% in two or three regions, and 15.6% in four or more regions (Table 2). As shown in Table 3, the shoulders (46.4%), lower back (44.0%), and neck (36.9%) were the most frequently affected anatomical regions. Among the 84 orthodontists who reported musculoskeletal complaints, 25 (29.8%) indicated that they had been obliged to cease work at some point during the previous 12 months as a result of these complaints, and 43 (51.2%) reported having experienced pain during the preceding 7 days (Table 4). Among those reporting work cessation, complaints of the knees and hand/wrist appeared relatively prominent compared with other anatomical regions. Half of the orthodontists with musculoskeletal complaints (*n* = 42) had sought medical or therapeutic assistance, most commonly from a physiotherapist or manual therapist (Table 5). In addition, 21 (25.0%) reported having used medication in relation to their musculoskeletal complaints.

**Table 2:** Prevalence of musculoskeletal complaints during the preceding 12 months among surveyed orthodontists.

	*n* (%)
Participants	115 (100%)
Any musculoskeletal complaint	84 (73.0%)
No musculoskeletal complaints	31 (27.0%)
Number of anatomical regions affected^a^	
One anatomical region	33 (28.7%)
Two anatomical regions	20 (17.4%)
Three anatomical regions	13 (11.3%)
Four anatomical regions	9 (7.8%)
Five anatomical regions	6 (5.2%)
Six anatomical regions	2 (1.7%)
Seven anatomical regions	1 (0.9%)

^a^Percentages for number of anatomical regions affected are calculated among all participants (*n* = 115).

**Table 3:** Musculoskeletal complaints during the preceding 12 months by anatomical region among orthodontists reporting complaints.

	*n* (%)
Participants with WMSDs	84 (100%)^a^
Neck	31 (36.9%)
Shoulder	39 (46.4%)
Left shoulder	12 (30.8%)^b^
Right shoulder	17 (43.6%)^b^
Both shoulders	10 (25.6%)^b^
Elbow	9 (10.7%)
Left elbow	3 (33.3%)^b^
Right elbow	6 (66.7%)^b^
Both elbow	0 (0%)^b^
Hand and wrist	20 (23.8%)
Left side	3 (15.0%)^b^
Right side	12 (60.0%)^b^
Both sides	5 (25.0%)^b^
Upper back	19 (22.6%)
Lower back	37 (44.0%)
Hips and thighs	13 (15.5%)
Knees	14 (16.7%)
Ankles and feet	15 (17.9%)

^a^Respondents could report complaints in multiple anatomical regions; therefore, percentages do not sum to 100%.

^b^Percentages for subcategories (left/right/both) are calculated within the corresponding anatomical region.

**Table 4:** Work cessation during the preceding 12 months and musculoskeletal complaints during the preceding 7 days among orthodontists reporting complaints.

Participants with WMSDs (*n* = 84)		
Work cessation during the preceding 12 months		
Anatomical region	Ceased work (*n* = 25, 29.8%)	Did not cease work (*n* = 59, 70.2%)
Neck	4 (16.0%)	17 (28.8%)
Shoulders	2 (8.0%)	20 (33.9%)
Elbows	2 (8.0%)	20 (33.9%)
Hand and wrist	5 (20.0%)	27 (45.8%)
Upper back	4 (16.0%)	15 (25.4%)
Lower back	3 (12.0%)	14 (23.7%)
Hips and thighs	2 (8.0%)	23 (39.0%)
Knees	7 (28.0%)	17 (28.8%)
Ankles and feet	5 (20.0%)	19 (32.2%)

Complaints during the preceding 7 days		
Anatomical region	Complaints (*n* = 43, 51.2%)	No complaints (*n* = 41, 48.8%)
Neck	12 (27.9%)	19 (46.3%)
Shoulders	14 (32.6%)	19 (46.3%)
Elbows	3 (7.0%)	6 (14.6%)
Hand and wrist	9 (20.9%)	11 (26.8%)
Upper back	5 (11.6%)	14 (34.1%)
Lower back	11 (25.6%)	19 (46.3%)
Hips and thighs	5 (11.6%)	8 (19.5%)
Knees	4 (9.3%)	10 (24.4%)
Ankles and feet	6 (14.0%)	9 (22.0%)

Percentages are calculated within each subgroup (e.g. cessation vs no cessation; complaints vs no complaints). Respondents could report complaints in multiple anatomical regions.

**Table 5:** Seeking medical or therapeutic care for musculoskeletal complaints and type of care sought among orthodontists reporting complaints.

	*n* (%)
Participants with WMSDs	84 (100%)
Sought medical or therapeutic care	
Yes	42 (50.0%)
No	42 (50.0%)
Type of care sought^a,b^	
Physiotherapy or manual therapy	31 (36.9%)
Chiropractor	4 (4.8%)
Surgery	4 (4.8%)
Medical doctor	2 (2.4%)
Other	6 (7.1%)
Medication	21 (25.0%)

^a^Percentages for type of care are calculated among orthodontists reporting musculoskeletal complaints (*n* = 84).

^b^Respondents could report multiple types of care; therefore, percentages for type of care do not sum to 100%.

### Burden from working conditions, stress, and private circumstances

Nearly one-quarter of the orthodontists (24.1%, *n* = 26) reported feeling seriously burdened by specific aspects of their work, including time pressure, workload, managing work in progress, administrative duties, continuing professional development, and adapting to laws and regulations. Conversely, 20.4% (*n* = 22) indicated that they were not, or not at all, burdened by their working conditions. The mean score on the ‘Working conditions’ scale was 33.4 ± 7.2, with observed scores ranging from 11.0 to 52.0 (possible range: 11.0–55.0). The majority of respondents (66.0%, *n* = 70) reported low levels of stress when asked whether they felt in control of important matters, personal issues, and professional tasks. Only 3.8% (*n* = 4) reported high levels of stress. The mean score on the ‘Stress’ scale was 16.2 ± 4.2, with a minimum of 8.0 and a maximum of 26.0 (possible range: 7.0–35.0) (Table 6).

**Table 6:** Distribution and descriptive statistics of the scales ‘Working conditions’, ‘Stress’, and ‘Private circumstances’ among surveyed orthodontists.

Burden	Working conditions (*n* = 108), *n* (%)^a^	Stress (*n* = 106), n (%)^b^	Private circumstances (*n* = 106), *n* (%)^c^
Very low	2 (1.9%)	10 (9.4%)	9 (8.5%)
Low	20 (18.5%)	60 (56.6%)	31 (29.2%)
Neutral	60 (55.6%)	32 (30.2%)	52 (49.1%)
High	23 (21.3%)	4 (3.8%)	13 (12.3%)
Very high	3 (2.8%)	0 (0.0%)	1 (0.9%)
Mean	33.4	16.2	13.5
Median	33.0	15.5	14.0
Mode	29.0	15.0	14.0
Standard deviation	7.2	4.2	4.1
Minimum	11.0	8.0	5.0
Maximum	52.0	26.0	24.0
Cronbach’s alpha	0.853	0.791	0.827

Percentages are based on the number of respondents with complete data for each scale.

^a^Very low (score 11–16), low (score 17–27), neutral (score 28–38), high (score 39–49), very high (score 50–55).

^b^Very low (score 7–10), low (score 11–17), neutral (score 18–24), high (score 25–31), very high (score 32–35).

^c^Very low (score 5–7), low (score 8–12), neutral (score 13–17), high (score 18–22), very high (score 23–25).

With regard to private circumstances, 49.1% (*n* = 42) of orthodontists scored neutrally on the ‘Private circumstances’ scale, while 37.7% (*n* = 40) reported that they were not, or not particularly, burdened by private circumstances. Despite the relatively low overall scale score, specific items that received comparatively higher scores concerned insufficient spare time, limited time for maintaining friendships, and limited time for caring for children and/or managing the household. The mean score on the ‘Private circumstances’ scale was 13.5 ± 4.1, with scores ranging from 5.0 to 24.0 (possible range: 5.0–25.0) (Table 6).

### Coping ability

Only 1.0% (*n* = 1) of orthodontists reported difficulty in coping with problems and incidents. In contrast, 52.4% (*n* = 55) indicated that they were somewhat able to cope with stressful situations, and 46.6% (*n* = 49) reported that they were reasonably able to do so. The mean score on the ‘Coping ability’ scale was 28.7 ± 3.4, with observed scores ranging from 20.0 to 37.0 (possible range: 8.0–40.0) (Table 7).

**Table 7:** Distribution and descriptive statistics of the scales ‘Coping ability’ and ‘Job satisfaction’ among surveyed orthodontists.

	Coping ability (*n* = 105), *n* (%)^a^	Job satisfaction (*n* = 104), *n* (%)^b^
Very low	0 (0.0%)	0 (0.0%)
Low	1 (1.0%)	4 (3.9%)
Neutral	55 (52.4%)	23 (22.1%)
High	37 (35.2%)	60 (57.7%)
Very high	12 (11.4%)	17 (16.3%)
Mean	28.7	38.3
Median	28.0	39.0
Mode	26.0	44.0
Standard deviation	3.4	6.5
Minimum	20.0	16.0
Maximum	37.0	50.0
Cronbach’s alpha	0.745	0.855

Percentages are based on the number of respondents with complete data for each scale.

^a^Very low (score 8–12), low (score 13–20), neutral (score 21–28), high (score 29–36), very high (score 37–40).

^b^Very low (score 10–14), low (score 15–24), neutral (score 25–34), high (score 35–44), very high (score 45–50).

### Job satisfaction

With regard to career goals, reflection on the decision to become an orthodontist, future plans, and the performance of their professional duties, the vast majority of respondents (74.0%, *n* = 77) reported being (very) satisfied with their job, whereas 3.9% (*n* = 4) indicated that they were not satisfied. The mean score on the ‘Job satisfaction’ scale was 38.3 ± 6.5, with observed scores ranging from 16.0 to 50.0 (possible range: 10.0–50.0) (Table 7). As shown in Table 8, bivariate analyses demonstrated that job satisfaction was negatively correlated with the number of WMSDs and with perceived burden from working conditions and stress. In contrast, job satisfaction was positively associated with practice ownership and coping ability. Multivariable linear regression analysis indicated that variation in job satisfaction could be partly explained (adjusted *R*^2^ = 0.252) by three characteristics: perceived burden from working conditions, coping ability, and practice ownership. Orthodontists who experienced less burden from their work and who reported greater coping ability were more satisfied with their job. In addition, practice ownership was positively associated with job satisfaction (Table 9).

**Table 8:** Bivariate associations between job satisfaction and selected personal and professional characteristics among surveyed orthodontists.

Variable	β	t	*P*
Sex (male/female)	0.010	0.104	.917
Age	0.129	1.131	.192
Family composition: single (yes/no)	−0.049	−0.493	.623
Family composition: children (yes/no)	−0.017	−0.173	.863
Regularly participating in sports (yes/no)	0.008	0.078	0.938
Number of WMSDs last 12 months	**−0**.**297**	**−3**.**140**	.**002**
WMSDs last 7 days (yes/no)	−0.122	−1.240	.218
Number of years practising orthodontics	0.146	1.495	.138
Private practice owner (yes/no)	**0**.**266**	**2**.**787**	.**006**
Number of treatment chairs	−0.046	−0.396	.693
Burdened by working conditions (yes/no)	**−0**.**405**	**−4**.**472**	**<.001**
Burdened by stress (yes/no)	**−0**.**212**	**−2**.**188**	.**031**
Burdened by private circumstances (yes/no)	−0.089	−0.900	.370
Coping ability	**0**.**198**	**2**.**041**	.**044**

Statistical significance was defined as *P* < .05. Bold values indicate statistically significant associations.

Cronbach’s alpha coefficients ≥ 0.70 were considered to indicate acceptable internal consistency.

β = standardized regression coefficient; t = t-statistic; *P* values derived from bivariate linear regression analyses.

**Table 9:** Multivariable associations between job satisfaction and relevant personal and professional characteristics of surveyed orthodontists.

Variable	β	t	*P*
Private practice owner	0.286	3.351	.**001**
Burdened by working conditions	−0.402	−4.698	**<.001**
Coping ability	0.177	2.073	**.041**

Statistical significance was defined as *P* < .05. Bold values indicate statistically significant associations.

Adjusted *R*^2^ = 0.252, indicating the proportion of variance in job satisfaction explained by the model.

β = standardized regression coefficient; t = t-statistic; *P* values derived from multivariable linear regression analysis.

## Discussion

The aim of this study was to investigate the extent to which orthodontists experience physical complaints and burdensome working conditions, to assess their level of job satisfaction, and to identify factors associated with job satisfaction. The findings indicate that a substantial majority of orthodontists report physical complaints. Not only was the overall prevalence high, but multiple anatomical regions were frequently affected, with the shoulders, neck, and lower back being the most commonly reported sites. In contrast, perceived burden from working conditions was more evenly distributed: some orthodontists reported being seriously burdened, whereas others reported no burden at all.

Although a higher number of physical complaints were negatively associated with job satisfaction, the strongest determinants of greater job satisfaction were more favourable working conditions, practice ownership, and higher coping ability. Nevertheless, these determinants accounted for only 25% of the variance in job satisfaction, indicating that additional factors not examined in this study are likely to contribute.

The present study demonstrated that WMSDs are highly prevalent among Dutch orthodontists, with almost three-quarters reporting such complaints. The shoulders, neck, and lower back were the most frequently affected regions. These findings are consistent with previous research among orthodontists, in which prevalence rates ranged from 72% to 83%, and the same anatomical regions were most commonly affected [14, 15, 27].

In accordance with previous studies among dentists and orthodontists, most respondents in this study reported low levels of occupational stress [5–7, 12, 18]. However, time pressure, workload, and administrative tasks were identified as the principal stressors, which accords with previous findings. Earlier studies also reported staff-related issues as important sources of stress [5, 6]. As staff-related items were not included in the present questionnaire, work-related stress may have been underestimated.

The majority of orthodontists reported high job satisfaction, consistent with previous studies demonstrating satisfaction rates between 71% and 90% [6, 20, 28, 29]. While the overall prevalence of job satisfaction is comparable with the literature, the determinants reported vary. Roth *et al.* (2003, 2004) [6, 20] found that quality of life, patient relationships, and professional respect enhanced job satisfaction among Canadian orthodontists, whereas occupational stress, practice management, and limited personal time reduced satisfaction. Onabolu *et al.* (2018) [28], in a study among orthodontic therapists in the UK, reported that remuneration and recognition for good work were associated with lower satisfaction, whereas relationships with colleagues contributed positively. Comparable prevalence rates of WMSDs have been reported in other occupations involving prolonged static postures, such as administrative workers performing computer-based tasks, although the distribution of affected anatomical regions may differ. Al-Junaid *et al.* (2017) [29], in a study conducted in the UK and Republic of Ireland, identified fewer working hours, greater control over the working day, effective management of domestic issues, and management of patient expectations as positive determinants.

In agreement with parts of the existing literature, the present study found that favourable working conditions, practice ownership, and higher coping ability were the strongest determinants of job satisfaction. In addition, the number of WMSDs and perceived stress were associated with job satisfaction in bivariate analyses, although these associations did not remain statistically significant in the multivariable model. The variation in findings across studies suggests that determinants of job satisfaction are not yet fully elucidated and warrant further investigation.

### Limitations

Several limitations should be acknowledged. First, survivor bias may be present, as orthodontists who had already left the profession were not included. Those who discontinued practice may have done so due to low job satisfaction or physical complaints, potentially leading to an overestimation of satisfaction levels. Secondly, although the response rate was acceptable (46.0%), nonresponse bias cannot be excluded. It is possible that orthodontists with lower job satisfaction were less likely to participate. Conversely, orthodontists experiencing musculoskeletal complaints may have been more inclined to respond, potentially leading to an overestimation of WMSD prevalence. In addition, some questionnaire items were incompletely answered, resulting in varying sample sizes across analyses (e.g. *n* = 108 for working conditions, *n* = 106 for stress and private circumstances, *n* = 105 for coping ability, and *n* = 104 for job satisfaction). Respondents with missing data were excluded from the relevant analyses (complete-case analysis), but retained for other parts of the study. Although this approach allowed the inclusion of as much available information as possible, it may have introduced bias if the missing data were not random. However, the proportion of missing data was relatively small and therefore unlikely to have materially affected the results. Thirdly, items relating to appreciation, financial factors, and staff-related issues were not included, despite evidence from other studies that these factors may influence job satisfaction. In addition, information on full-time versus part-time working status was not collected. This may influence workload, exposure to physical strain, and perceived stress, and should be considered in future research. Finally, as the sample consisted solely of Dutch orthodontists, the findings may primarily be generalizable to orthodontists practising in comparable Western healthcare systems.

### Recommendations

This study provides insight into job satisfaction and its associated factors among orthodontists. The findings may contribute to awareness, prevention, and management of physical and psychological complaints in professional practice. The results may also be relevant to other stakeholders, including physicians, psychologists, and insurance providers.

Future research should aim to include orthodontists who have left the profession, as well as incorporate variables relating to appreciation, financial remuneration, and staff-related factors. In the present study, physical complaints were assessed quantitatively; qualitative research may provide deeper understanding of the nature and impact of these complaints. Furthermore, the methodological approach used here could be applied to other oral healthcare professionals, such as orthodontic assistants, dentists, dental hygienists, and dental assistants.

## Conclusion

Job satisfaction among Dutch orthodontists appears to be high. In this cross-sectional study, greater job satisfaction was most strongly associated with more favourable working conditions, practice ownership, and higher coping ability. Lower perceived stress and fewer work-related musculoskeletal complaints were associated with job satisfaction in unadjusted analyses, but did not remain independent predictors in the multivariable model. As the identified variables explained only a quarter of the variance in job satisfaction, additional factors not examined in this study are likely to contribute to professional satisfaction among orthodontists. Further research is warranted to explore these determinants in greater depth.

## Data Availability

All relevant data are within the manuscript. The data underlying this article will be shared on reasonable request to the corresponding author.
